# Supplementary figures and images for: MORC2 mediates transcriptional regulation through liquid-liquid phase separation
Source: eLife. 2026 May 20;14:RP108479. doi: 10.7554/eLife.108479 (PMC13189624; doi:10.7554/eLife.108479)

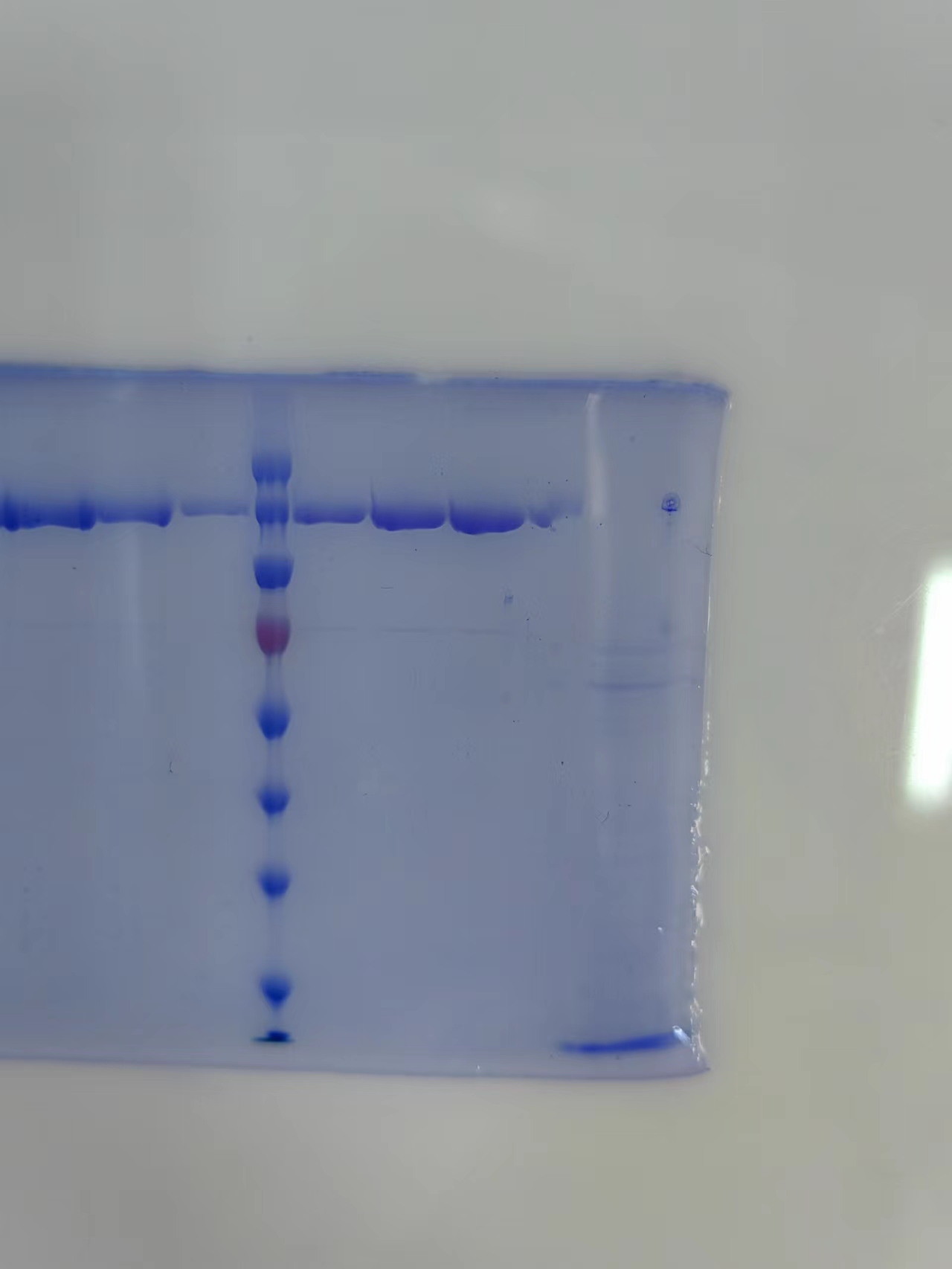

Supplement: Figure 1—source data 1. [file elife-108479-fig1-data1.zip › Figure 1-source data 1/MORC2FL-0930.tif]

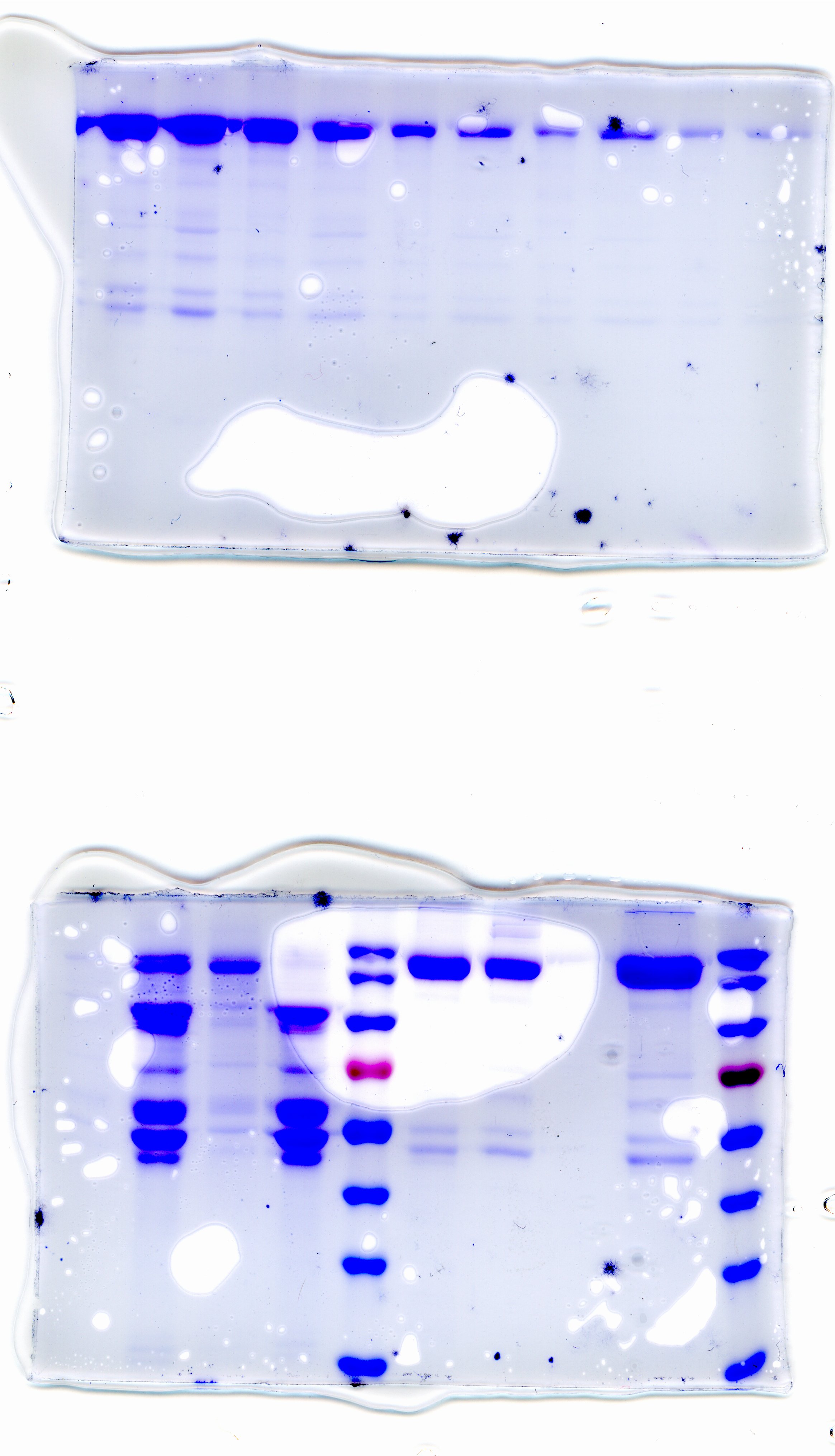

Supplement: Figure 2—source data 1. [file elife-108479-fig2-data1.zip › EGFP-MORC2FL-20221217_17305198.tif]

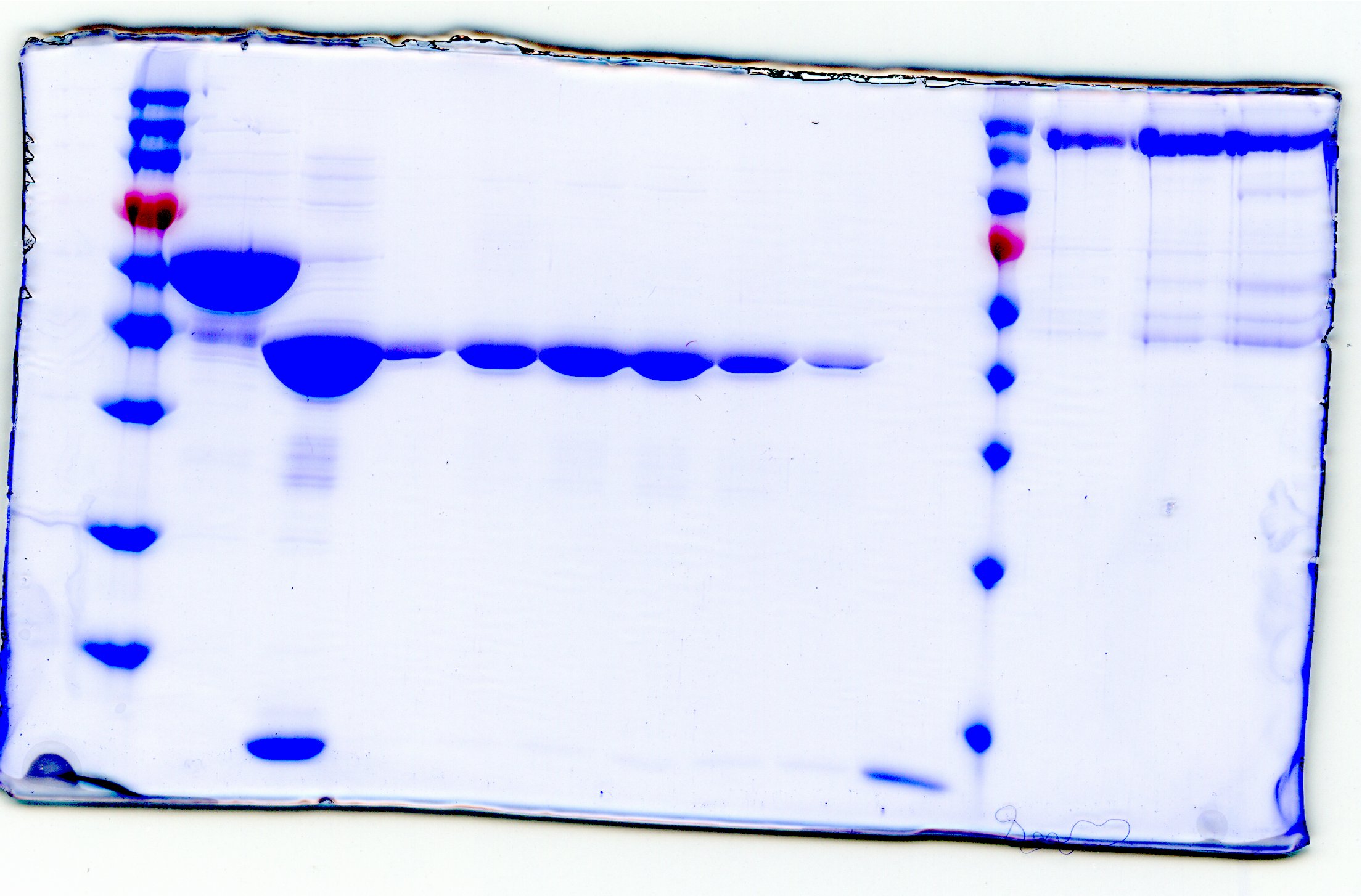

Supplement: Figure 2—source data 1. [file elife-108479-fig2-data1.zip › EGFP-MPRC2FL-20221129_12130550.tif]

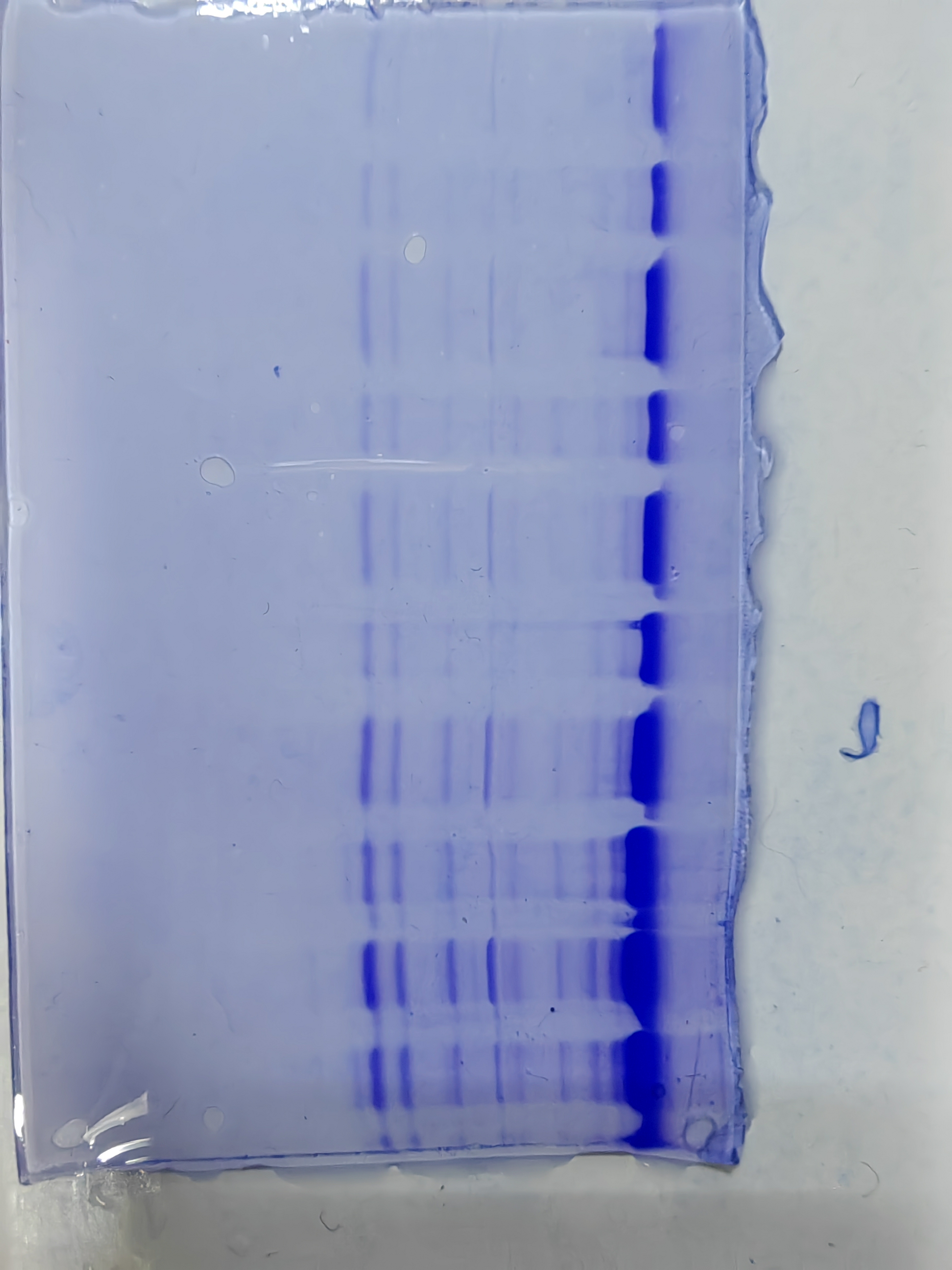

Supplement: Figure 2—figure supplement 1—source data 1. [file elife-108479-fig2-figsupp1-data1.zip › EGFP-MORC2_20221206191724.jpg]

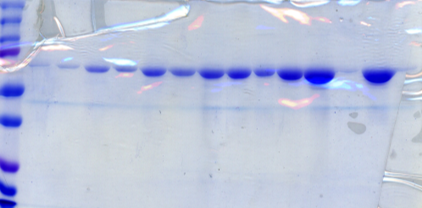

Supplement: Figure 3—source data 1. [file elife-108479-fig3-data1.zip › Cy3-MORC2-C-1.tif]

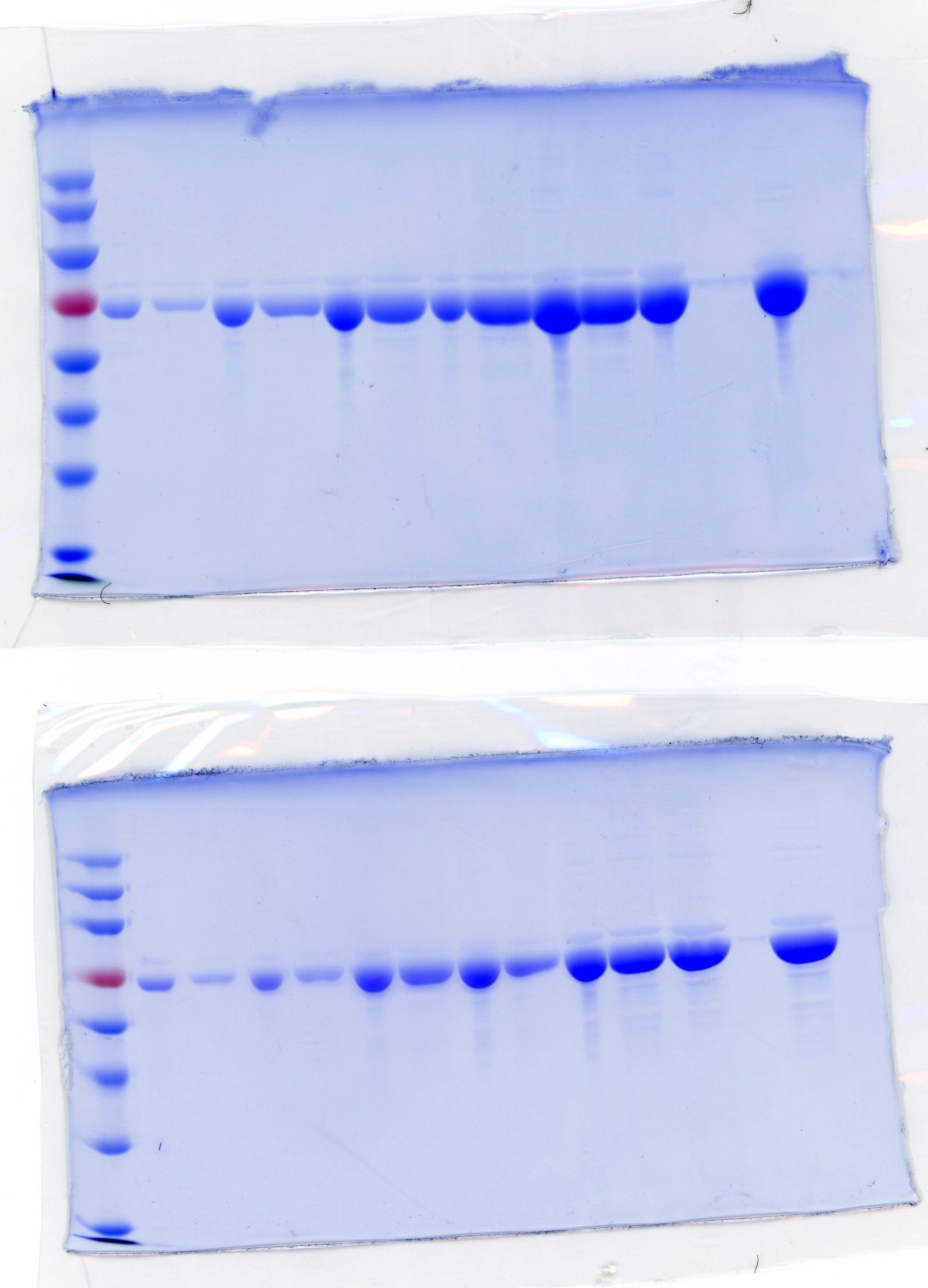

Supplement: Figure 3—source data 1. [file elife-108479-fig3-data1.zip › Cy3-MORC2-C-2.tif]

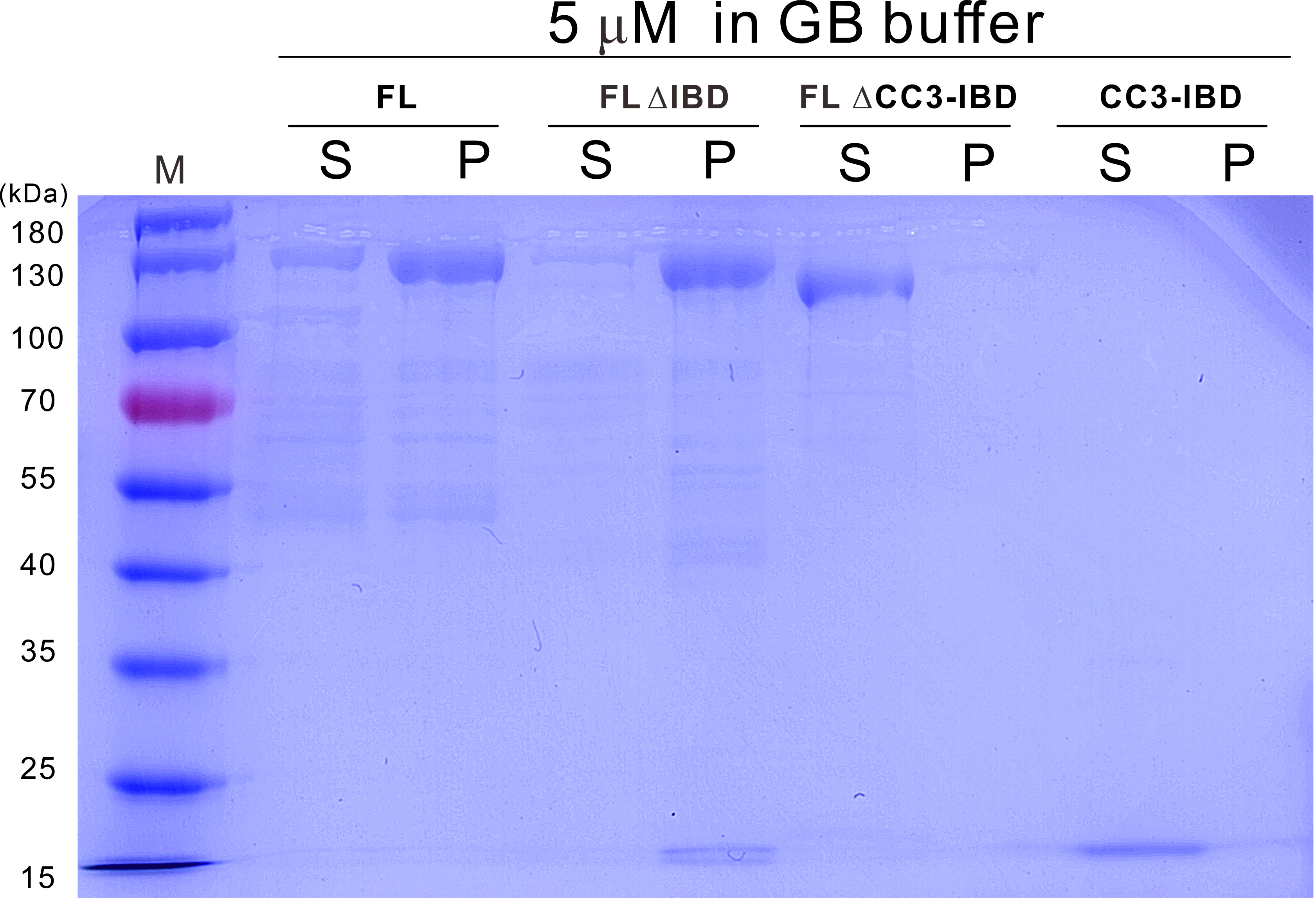

Supplement: Figure 3—figure supplement 3—source data 1. [file elife-108479-fig3-figsupp3-data1.zip › 1-1003 and 1004-1032.tif]

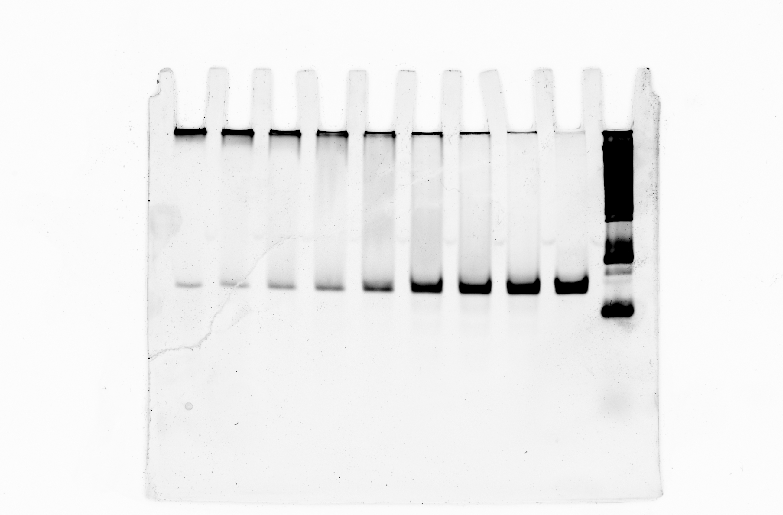

Supplement: Figure 5—source data 1. [file elife-108479-fig5-data1.zip › Figure 5a.tif]

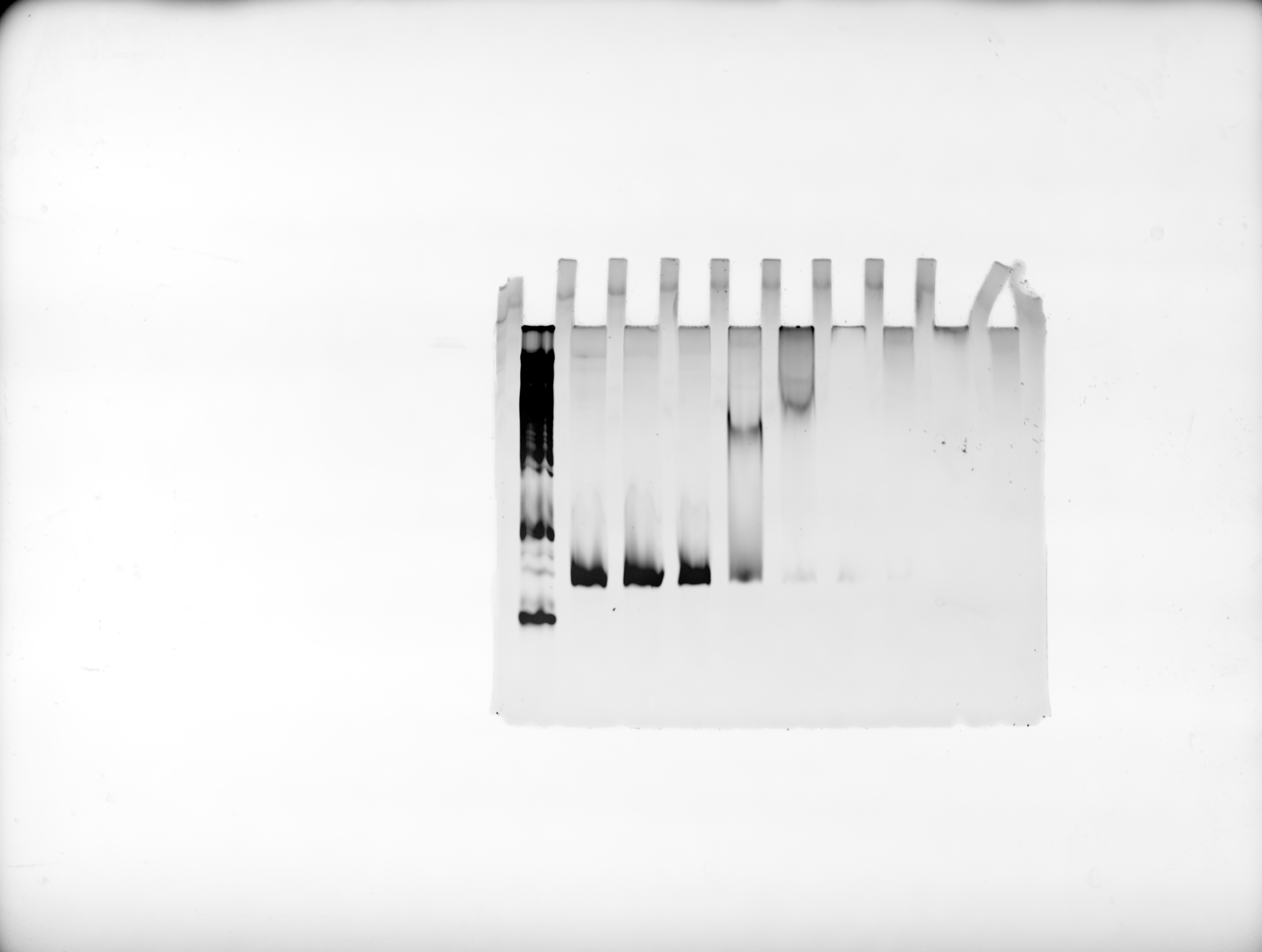

Supplement: Figure 5—source data 1. [file elife-108479-fig5-data1.zip › Figure 5b.tif]

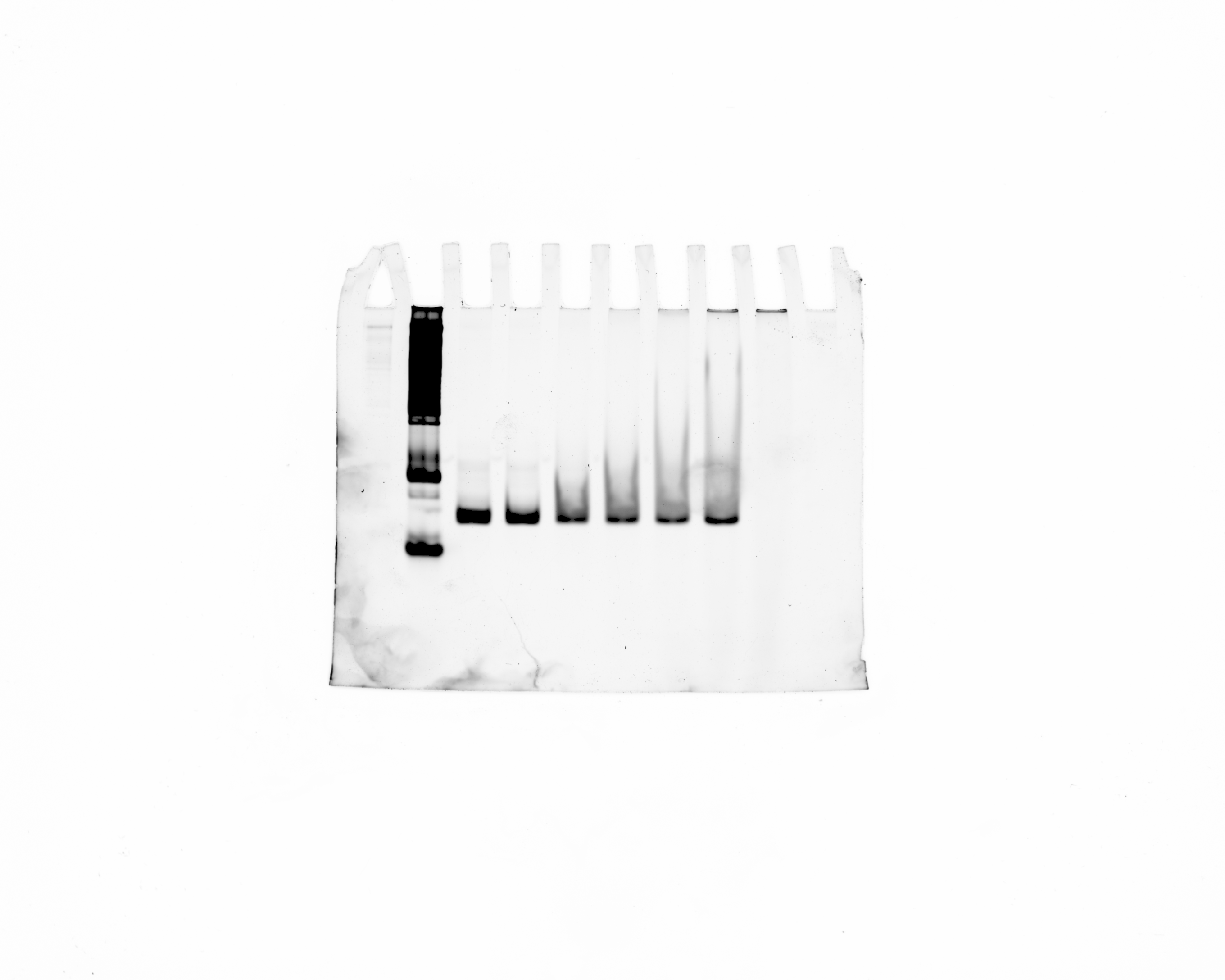

Supplement: Figure 5—source data 1. [file elife-108479-fig5-data1.zip › Figure 5c.tif]

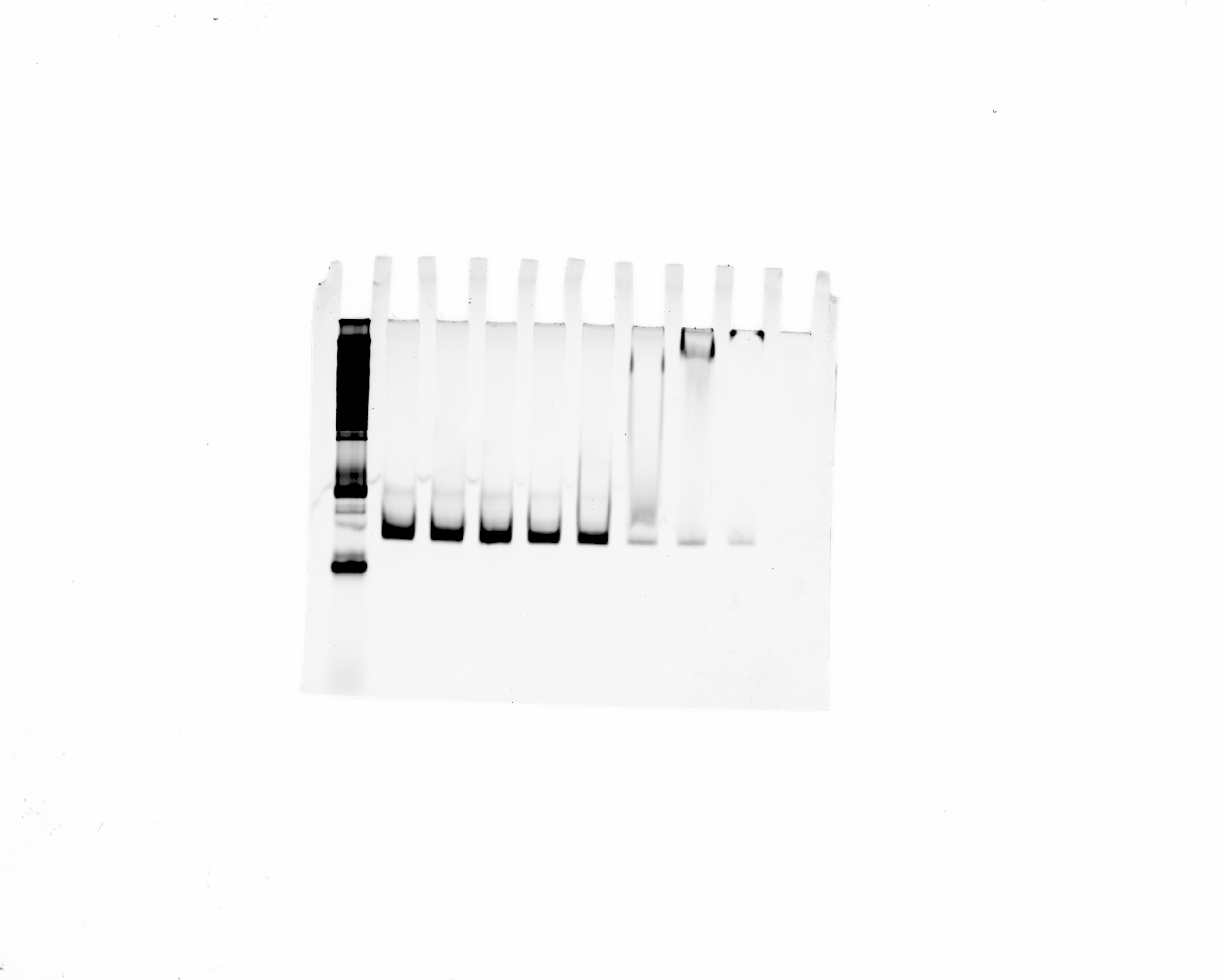

Supplement: Figure 5—source data 1. [file elife-108479-fig5-data1.zip › Figure 5d.tif]

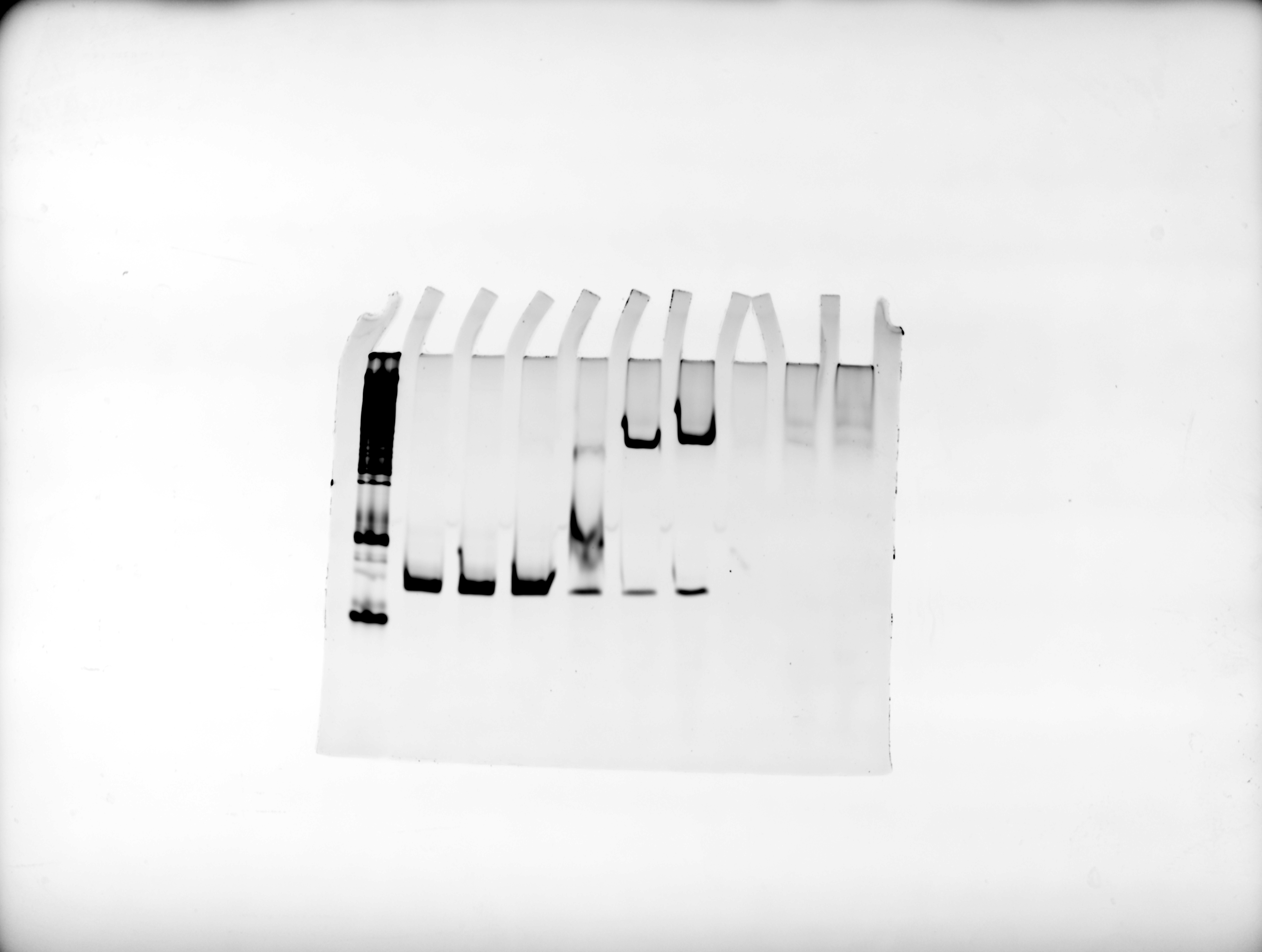

Supplement: Figure 5—source data 1. [file elife-108479-fig5-data1.zip › Figure 5e.tif]

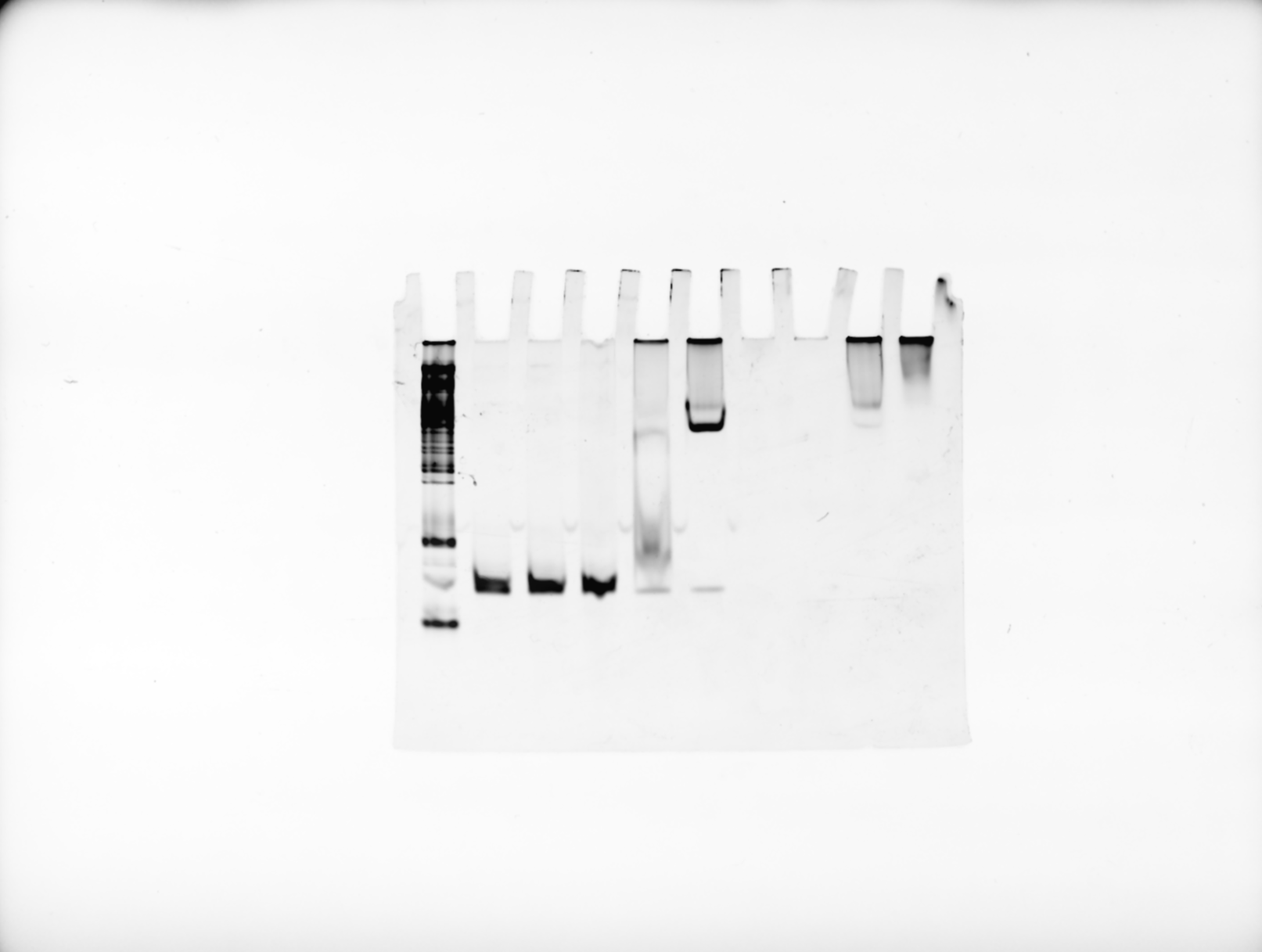

Supplement: Figure 5—source data 1. [file elife-108479-fig5-data1.zip › Figure 5f.tif]

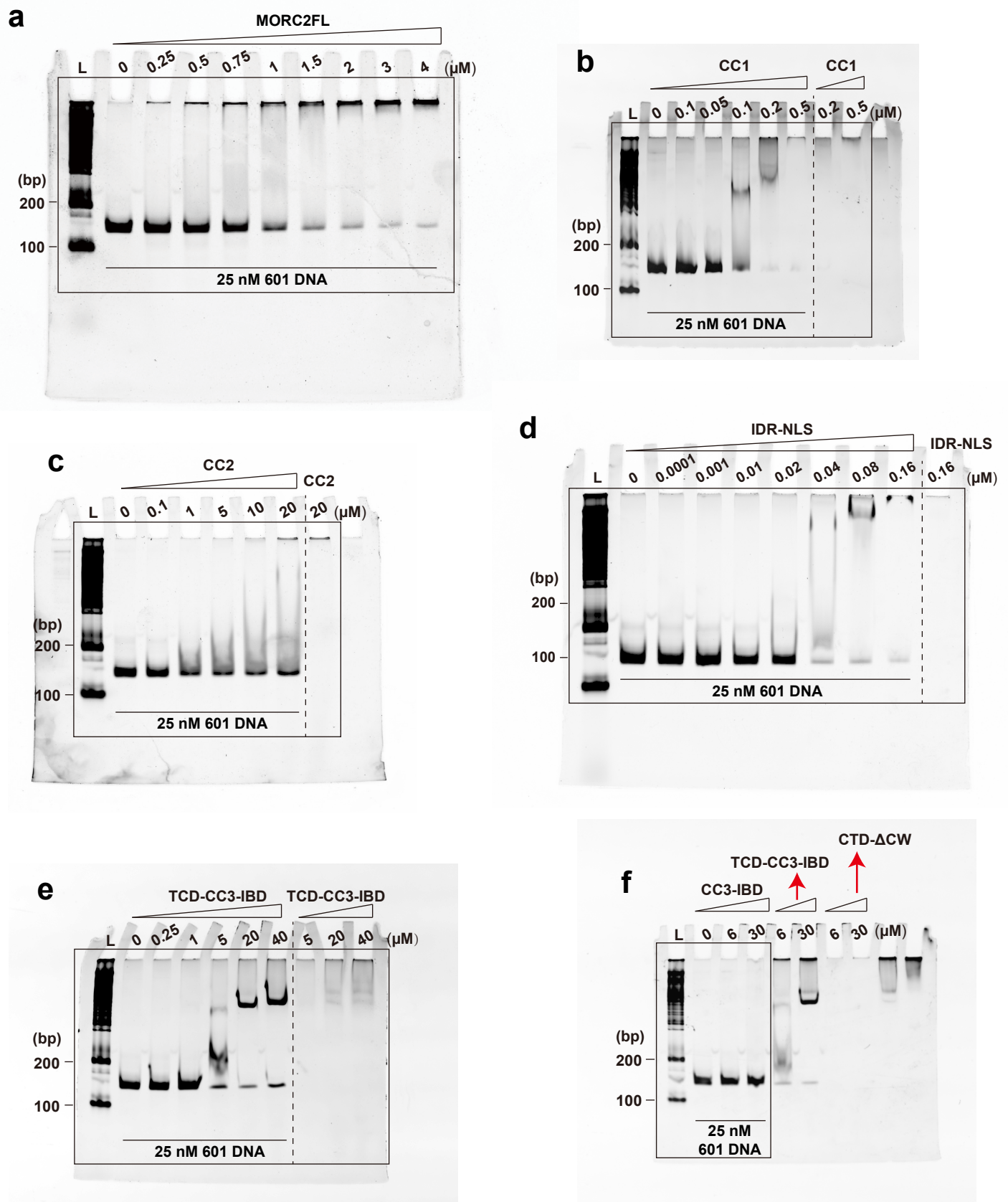

**Figure 5-Source data 1** Original gels corresponding to Figure 5, panel a-f

Supplement: Figure 5—source data 2. [file elife-108479-fig5-data2.zip › Figure 5-Source data 1.pdf]
