# Supplementary material for: MORC2 mediates transcriptional regulation through liquid-liquid phase separation
Source: eLife. 2026 May 20;14:RP108479. doi: 10.7554/eLife.108479 (PMC13189624; doi:10.7554/eLife.108479)
Supplement: Figure 1—source data 2. [file elife-108479-fig1-data2.zip › Figure 1-source data 2/Figure 1—source data 2.pdf]

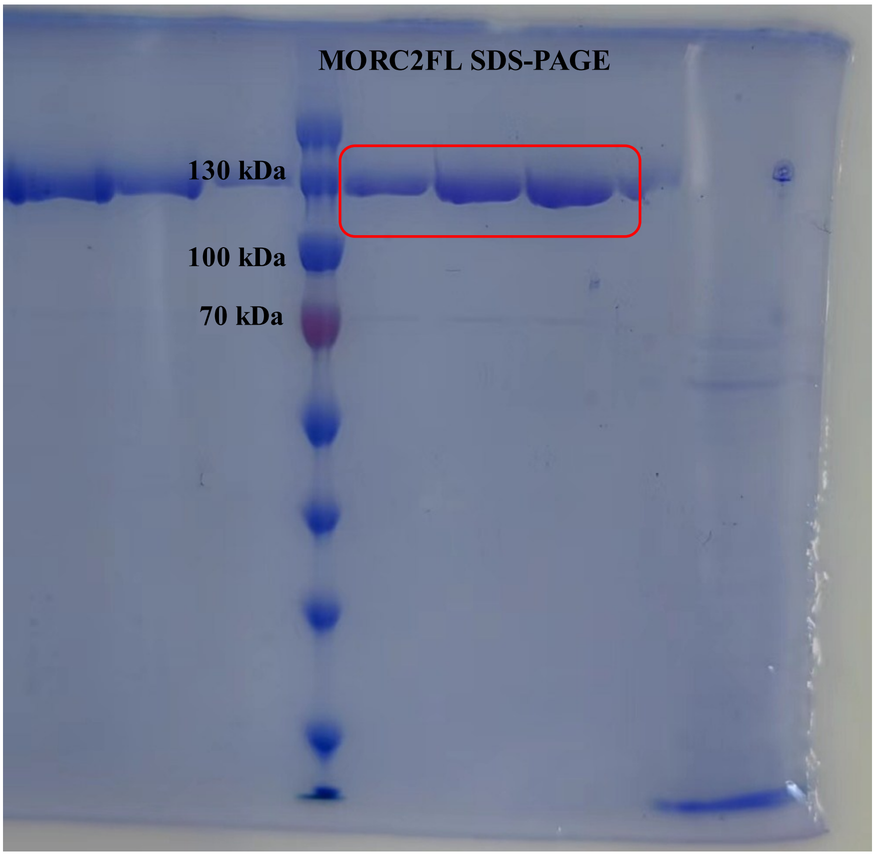


**Figure 1, Source Data 2.** Original SDS-PAGE gel corresponding to Figure 1b. Rainbow molecular weight markers were used. The lane utilized in Figure 1b is outlined in a red rectangular box.
