# Supplementary material for: MORC2 mediates transcriptional regulation through liquid-liquid phase separation
Source: eLife. 2026 May 20;14:RP108479. doi: 10.7554/eLife.108479 (PMC13189624; doi:10.7554/eLife.108479)
Supplement: Figure 2—source data 2. [file elife-108479-fig2-data2.zip › Figure 2—source data 2.pdf]

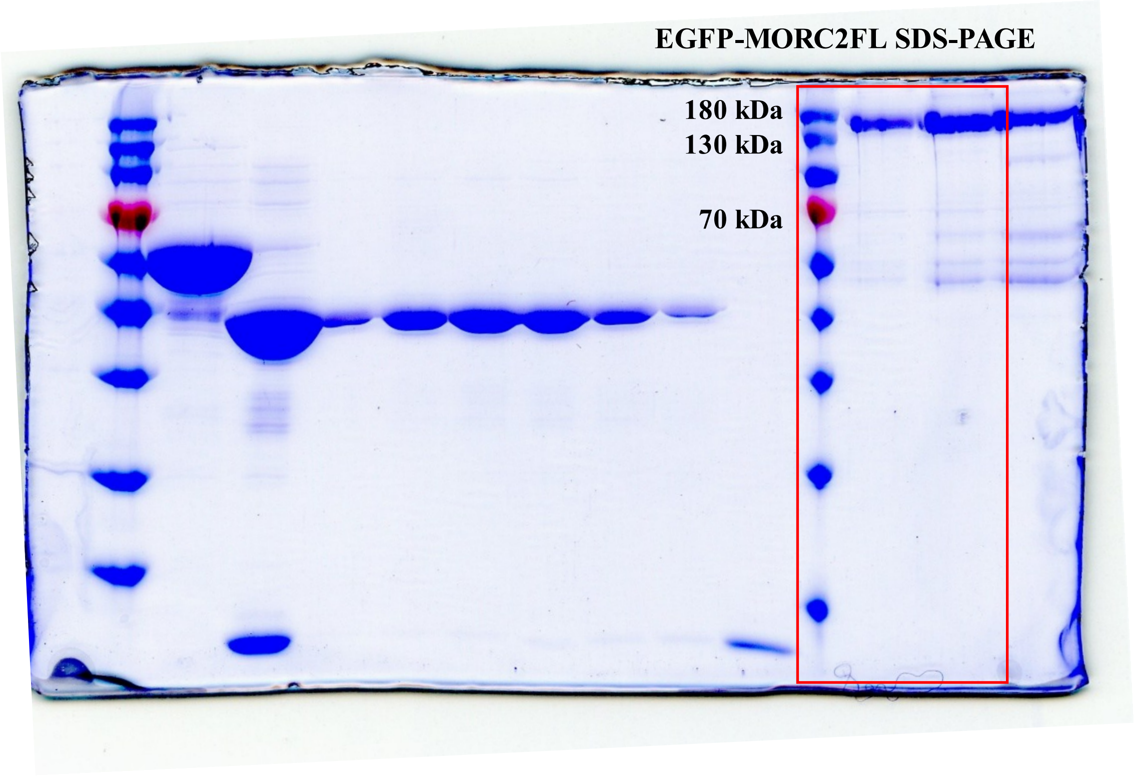

EGFP-MORC2FL Spin down SDS-PAGE

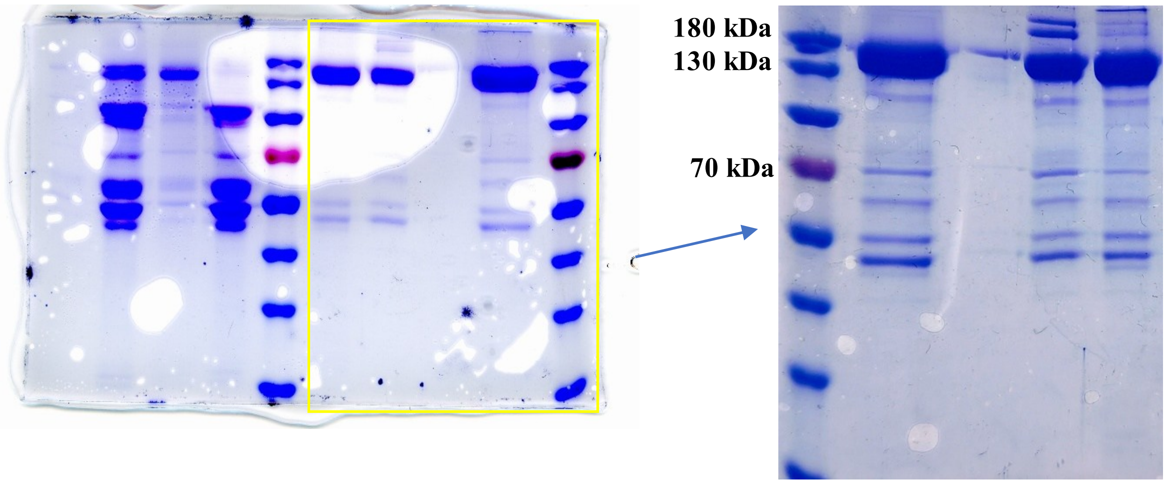

Figure 2, Source Data 2. Original SDS-PAGE gel corresponding to Figure 2a and 2b. Rainbow molecular weight markers were used. The lane used in Figure 2a is outlined in a red rectangular box, and another relevant lane is marked with a yellow rectangular box. Panel b was rotated 180° and only the target region was captured by local imaging.
