# Supplementary material for: MORC2 mediates transcriptional regulation through liquid-liquid phase separation
Source: eLife. 2026 May 20;14:RP108479. doi: 10.7554/eLife.108479 (PMC13189624; doi:10.7554/eLife.108479)
Supplement: Figure 2—figure supplement 1—source data 2. [file elife-108479-fig2-figsupp1-data2.zip › Figure 2—figure supplement1-source data 2.pdf]

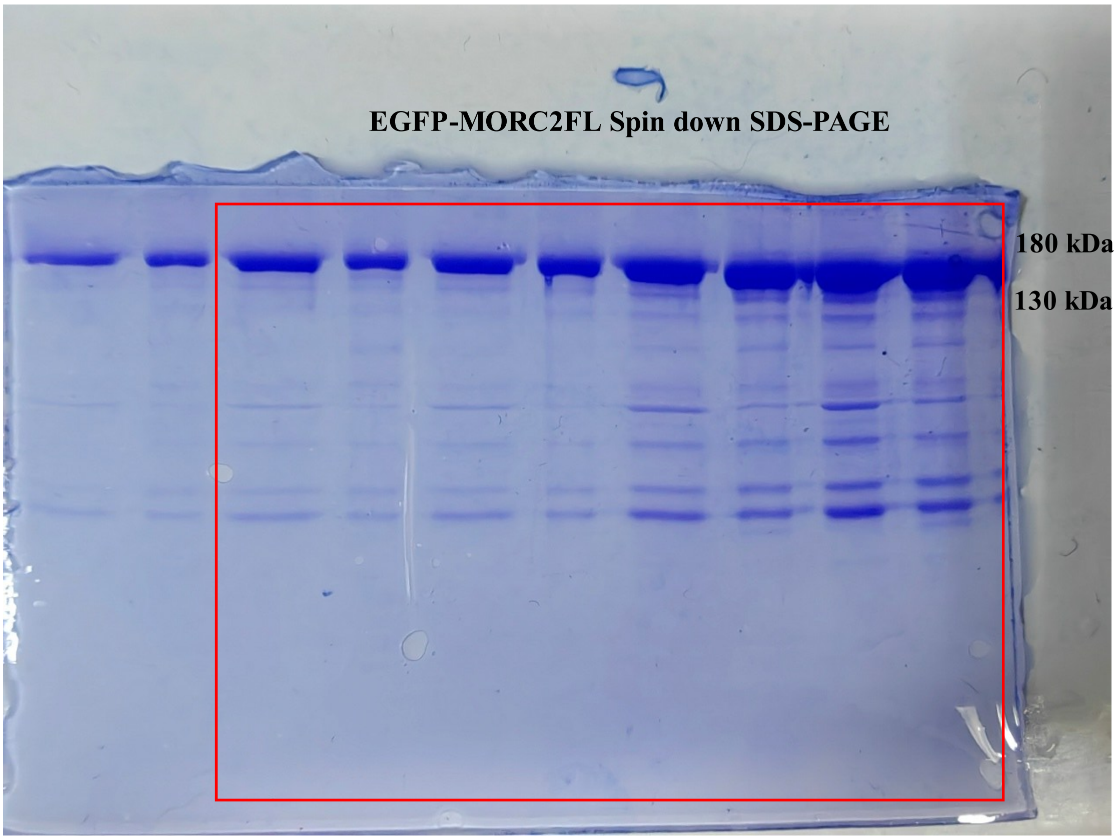

Figure 2-figure supplement 1, Source data 2. Original SDS-PAGE gel corresponding to Figure 2-figure supplement 1b. Molecular weight was consistently estimated using parallel SDS-PAGE gels prepared in the same batch. The lane utilized is outlined in a red rectangular box.
