# Supplementary material for: MORC2 mediates transcriptional regulation through liquid-liquid phase separation
Source: eLife. 2026 May 20;14:RP108479. doi: 10.7554/eLife.108479 (PMC13189624; doi:10.7554/eLife.108479)
Supplement: Figure 3—source data 2. [file elife-108479-fig3-data2.zip › Figure 3-source data 2/Figure 3—source data 2.pdf]

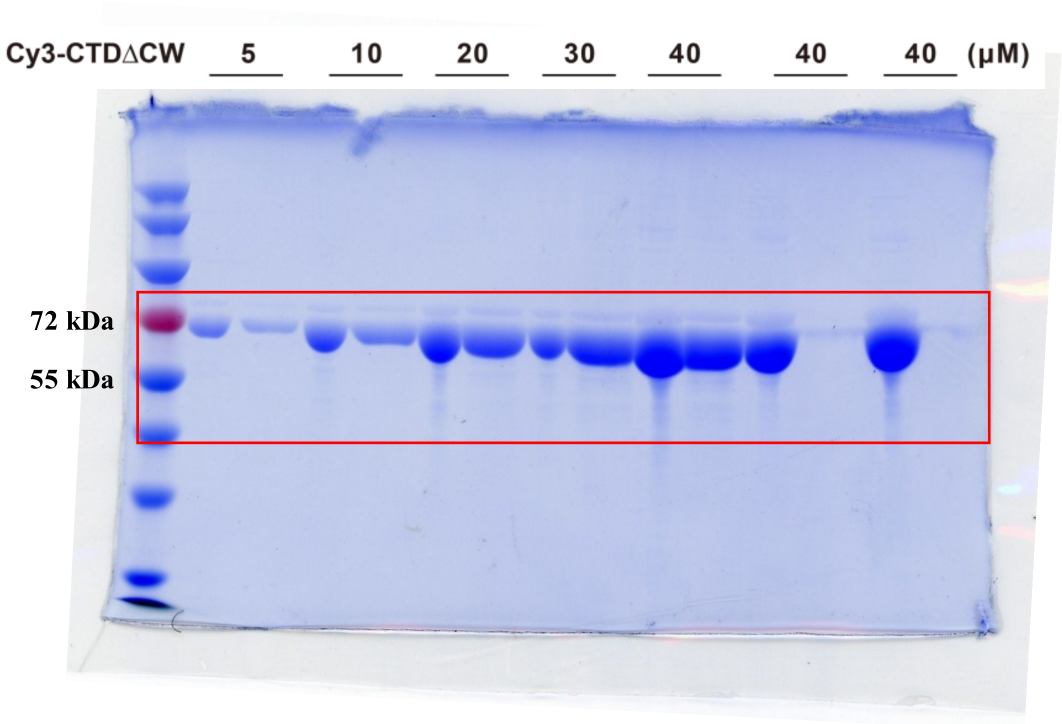

Figure 3, Source Data 2. Original SDS-PAGE gel corresponding to Figure 3d. Rainbow molecular weight markers were used. The lane shown in Figure 3d is outlined in a red rectangular box. The first lane represents the supernatant sample, and the second lane represents the pellet sample. Three independent experimental repeats are presented in Source Data 1.
