# Supplementary material for: MORC2 mediates transcriptional regulation through liquid-liquid phase separation
Source: eLife. 2026 May 20;14:RP108479. doi: 10.7554/eLife.108479 (PMC13189624; doi:10.7554/eLife.108479)
Supplement: Figure 3—figure supplement 3—source data 2. [file elife-108479-fig3-figsupp3-data2.zip › Figure 3—figure supplement 3-source data 2.pdf]

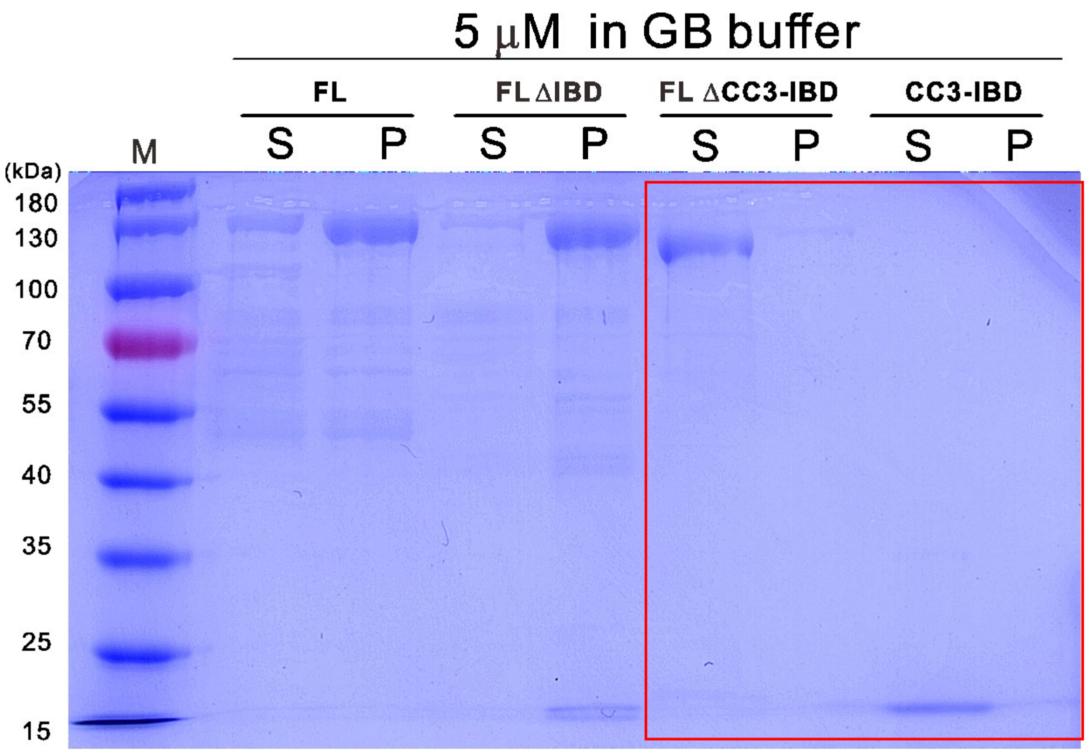

Figure 3-figure supplement 3, Source data 2. Original SDS-PAGE gel corresponding to Figure 3-figure supplement 3a. Rainbow molecular weight markers were used. The lane utilized in Figure 3-figure supplement 3a is outlined in a red rectangular box.
S: supernatant sample and P: pellet sample.
