# Supplementary material for: MORC2 mediates transcriptional regulation through liquid-liquid phase separation
Source: eLife. 2026 May 20;14:RP108479. doi: 10.7554/eLife.108479 (PMC13189624; doi:10.7554/eLife.108479)
Supplement: Supplementary file 1. [file elife-108479-supp1.doc]

**Table 1**

Statistics of X-ray Crystallographic Data Collection and Model refinement

| | **Data collection** |  | | --- | --- | | Data sets | MORC2-CC3 | | Space group | *I41* | | Wavelength (Å) | 0.97915 | | Unit Cell Parameters (Å) | a=130.253, b=130.253, c= 40.895  α=β=γ=90° | | Resolution range (Å) | 50-3.05 (3.10-3.05) | | No. of unique reflections | 6774 (333) | | Redundancy | 12.6 (10.5) | | I/σ | 25.897 (2.600) | | Completeness (%) | 100.0 (100.0) | | R_merge_ ^a^ (%) | 12.0 (121.4) | | CC_1/2_ (last resolution shell) ^b^ | 0.703 | |  |
| --- | --- | --- | --- | --- | --- | --- | --- | --- | --- | --- | --- | --- | --- | --- | --- | --- | --- | --- | --- | --- | --- | --- | --- | --- | --- |
| **Structure refinement** |  |
| | Resolution (Å) | 32.56-3.10 | | --- | --- | | R_cryst_ c/R_free_ ^d^ (%) | 19.81 / 24.80 | | Rmsd bonds (Å) / angles (°) | 0.004 / 0.627 | | Average B factor (Å^2^) ^e^ | 57.55 | | No. of atoms |  | | Protein atoms | 1543 | | Water | 6 | | Ligands | 0 | | Ramachandran plot regions ^e^ |  | | Favored (%) | 98.99 | | Allowed (%) | 1.01 | | Outliers (%) | 0 | |  |

Numbers in parentheses represent the value for the highest resolution shell.

(**a**) R_merge_ = Σ |*I_i_* - <*I*>| / Σ*I_i_*, where *I_i_* is the intensity of measured reflection and <*I*> is the mean intensity of all symmetry-related reflections.

(**b**) CC_1/2_ were defined by Karplus and Diederichs.

(**c**) R_cryst_=Σ||*F*_calc_| – |*F*_obs_||/Σ*F*_obs_, where *F*_obs_ and *F*_calc_ are observed and calculated structure factors.

(**d**) R_free_= Σ_T_||*F*_calc_| – |*F*_obs_||/Σ*F*_obs_, where T is a test data set of about 5% of the total unique reflections randomly chosen and set aside prior to refinement.

(**e**) B factors and Ramachandran plot statistics are calculated using MOLPROBITY.
