# Supplementary material for: MORC2 mediates transcriptional regulation through liquid-liquid phase separation
Source: eLife. 2026 May 20;14:RP108479. doi: 10.7554/eLife.108479 (PMC13189624; doi:10.7554/eLife.108479)
Supplement: Supplementary file 2. [file elife-108479-supp2.doc]

**Table 2**

Summary of MORC2 protein constructs used in this study, including expression systems, extinction coefficients, A260/280 ratios, and corresponding experimental applications.

| **MORC2 fragment *in vitro*** | **Expression system** | **Ext. coefficient** | **A260/280 ratios** | **Experimental applications** |
| --- | --- | --- | --- | --- |
| **MORC2FL (1-1032)** | ***E. coli*** | 88240 | 0.59 | EMSA, Agarose gel analysis (AGA) |
| **CTD (473-1032)** | *E. coli* | 46410 | 0.71 | EMSA;  Phase separation |
| **CTD∆CW (537-1032)** | *E. coli* | 26930 | 0.58 | EMSA;  Phase separation;  FPLC-MALS |
| **TCD-CC3-IBD (744-1032)** | *E. coli* | 26930 | 0.67 | EMSA; FPLC-MALS |
| **CC3-IBD (901-1032)** | *E. coli* | 7450 | 0.51 | EMSA; FPLC-MALS, AGA |
| **CC1 (279-362)** | *E. coli* | 1490 | 0.53 | EMSA |
| **CC2 (551-603)** | *E. coli* | 0 | ND | EMSA |
| **CC3 (901-1003)** | *E. coli* | 5960 | 0.49 | FPLC-MALS NMR; Structure determination, AGA |
| **IDR (593-735)** | *E. coli* | 0 | ND | EMSA |
| **IBD (1004-1032)** | *E. coli* | 1490 | 0.58 | NMR |
| **IDRa (593-643)** | *E. coli* | 0 | ND | NMR |
| **IDRb (644-694)** | *E. coli* | 0 | ND | NMR |
| **IDRc (695-735)** | *E. coli* | 0 | ND | NMR |
| **Trx-CC3** | *E. coli* | 21555 | 0.6 | FPLC-MALS |
| **Trx-CC3^trun^_901-975** | *E. coli* | 14105 | 0.58 | FPLC-MALS |
| **Trx-CC3-IBD_L915Q** | *E. coli* | 21555 | 0.91 | FPLC-MALS |
| **Trx-CC3-IBD_F922Q** | *E. coli* | 21555 | 0.59 | FPLC-MALS |
| **Trx-CC3-IBD_L911Q** | *E. coli* | 21555 | 0.6 | FPLC-MALS |
| **Trx-CC3-IBD_I908Q** | *E. coli* | 21555 | 0.6 | FPLC-MALS |
| **Trx-CC3-IBD_F951Q** | *E. coli* | 21555 | 0.54 | FPLC-MALS |
| **Trx-CC3-IBD_Y921A** | *E. coli* | 20065 | 0.56 | FPLC-MALS |
| **Trx-CC3-IBD_F954Q** | *E. coli* | 21555 | 0.53 | FPLC-MALS |
| **Trx-CC3-IBD_L958Q** | *E. coli* | 21555 | 0.58 | FPLC-MALS |
| **MORC2FL (1-1032)** | **HEK293F** | 88240 | 0.6 | FPLC-MALS;  Phase separation; FP; ATPase activity, AGA |
| **MORC2∆CC3-IBD (1-900)** | HEK293F | 80790 | 0.56 | FPLC-MALS |
| **MORC2_N39A** | HEK293F | 88240 | 0.79 | FP; ATPase activity |
| **MORC2_R252W** | HEK293F | 93740 | 0.66 | FP; ATPase activity |
| **MORC2_Q400R** | HEK293F | 88240 | 0.65 | FP; ATPase activity |
| **MORC2_D466N** | HEK293F | 88240 | 0.58 | FP; ATPase activity |
| **MORC2_S87L** | HEK293F | 88240 | 0.75 | FP; ATPase activity |
| **MORC2_S218L** | HEK293F | 88240 | 0.72 | FP; ATPase activity |
| **MORC2_F256L** | HEK293F | 88240 | 0.6 | FP; ATPase activity |
| **MORC2_R266A** | HEK293F | 88240 | 0.75 | FP; ATPase activity |
| **MORC2_T424R** | HEK293F | 88240 | 0.55 | FP; ATPase activity |
| **EGFP-MORC2FL** | HEK293F | 110130 | 0.59 | Phase separation;  FRAP, AGA |
